# Ciprofloxacin-Loaded Spray-Dried Lactose Particles: Formulation Optimization and Antibacterial Efficacy

**DOI:** 10.3390/pharmaceutics17030392

**Published:** 2025-03-20

**Authors:** Sai Liu, Simon Gaisford, Gareth R. Williams

**Affiliations:** UCL School of Pharmacy, University College London, 29–39 Brunswick Square, London WC1N 1AX, UK; sai.liu.19@ucl.ac.uk (S.L.); s.gaisford@ucl.ac.uk (S.G.)

**Keywords:** antimicrobial, particle, spray drying, ciprofloxacin

## Abstract

**Background/Objectives**: Bacterial infections in the oral cavity and outer ear require effective and targeted drug delivery systems. This study details the production of drug-loaded lactose microparticles, with the aim of creating antibiotic formulations for ultimate use in combatting oral and outer ear bacterial infections. **Methods**: Lactose particles were prepared via spray drying and optimized with varying ciprofloxacin (cipro) loadings to maximize the drug content. The particles were characterized to evaluate their performance in terms of physicochemical properties, drug-loading efficiency, drug-release kinetics, and antibacterial activity. **Results**: The resulting particles exhibited spherical morphology, efficient cipro loading (in the range of 1.1−52.9% *w*/*w*) and rapid cipro release within 5 h (achieving 70−81% release). In addition, they demonstrated effective concentration-dependent antibacterial activity against gram-positive *Staphylococcus aureus* and gram-negative *Pseudomonas aeruginosa*, with bacterial growth effectively inhibited for more than 24 h when particle concentrations reached the minimum inhibitory concentration. **Conclusions**: These findings highlight the potential of spray-dried cipro loaded lactose particles as an efficient approach for localized antibacterial treatment, offering a promising solution for managing bacterial infections in the oral cavity and outer ear.

## 1. Introduction

Oral and outer ear bacterial infections represent major health challenges, contributing to significant morbidity and imposing a substantial economic burden on healthcare systems [1,2]. *Staphylococcus aureus* (*S. aureus*, a gram-positive bacterium) and *Pseudomonas aeruginosa* (*P. aeruginosa*, a gram-negative bacterium) are the most prevalent pathogens responsible for these infections [3,4]. Typically, *S. aureus* tends to initiate colonization, correlating with initial declines in host function and inflammation [5], whereas *P. aeruginosa* exerts a more prolonged and severe inflammatory response, leading to greater host damage [6].

**Figure 1:**
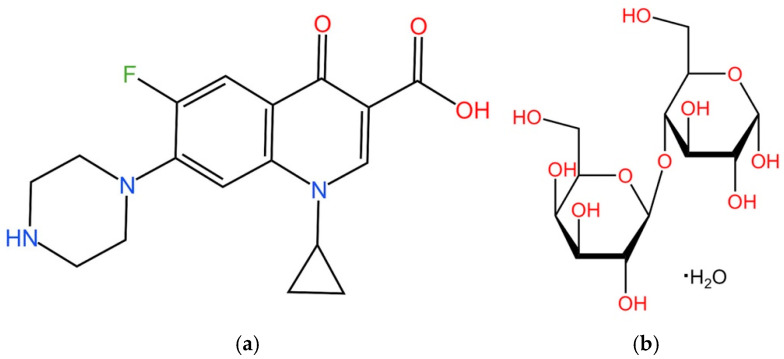
(**a**) Chemical structure of cipro and (**b**) chemical structure of LM (drawn in KingDraw version 3.5.8).

In the fight against oral and outer ear bacterial infections, antibiotics, including ciprofloxacin, amoxicillin, clindamycin, and metronidazole, are widely used [7,8]. Clinical studies indicate that antibiotics not only reduce the burden of infections but also can delay the onset of superinfections caused by gram-negative bacteria such as *P. aeruginosa* [9]. Among the approved antibiotics, ciprofloxacin (cipro), a fluoroquinolone antibiotic, is of particular importance (Figure 1a). Its widespread exploration stems not only from its broad-spectrum antibacterial properties but also its low minimal inhibitory concentration (MIC, generally less than 1 µg/mL) against both *S. aureus* and *P. aeruginosa* [10,11,12]. Research into the antibacterial effects of cipro dates back to 1984 [13,14,15], and it primarily achieves therapeutic effects by inhibiting bacterial DNA gyrase and topoisomerase IV, thereby disrupting DNA replication and leading to bacterial death [16].

However, cipro suffers from the disadvantages of both poor solubility at the neutral pH typical of physiological fluids and low permeability [17]. Despite its low MIC, biofilm formation by *P. aeruginosa* and *S. aureus* can complicate treatment [18,19]. Therefore, higher local drug concentrations may be necessary to overcome the barrier posed by biofilms and ensure therapeutic efficacy. Hence, there is a pressing need to devise effective strategies for its delivery. Spray drying (SD) provides an effective approach to enhance the solubility and dissolution rate of cipro [20]. By producing fine particles with a high surface-to-volume ratio, spray drying increases the surface area of cipro-loaded particles, facilitating faster dissolution and absorption—key factors for improving bioavailability in localized treatments [21]. Additionally, the spray-drying process transforms cipro into an amorphous form within the carrier matrix [22]. Amorphous drugs generally exhibit superior solubility compared to their crystalline counterparts due to the absence of an ordered molecular arrangement, enabling faster dissolution and overcoming solubility issues at physiological pH [22]. SD involves dispersing a solution into small droplets via an atomizer. These droplets are then swiftly dried within a drying chamber, facilitated by the high surface area of the droplets and a stream of hot gas. Subsequently, the resultant particles are transported into a cyclone and collected [20,21]. Spray drying is cost-effective and scalable [22] and can be used for processing a wide range of active ingredients, including antibiotics [23]. This versatility has led to the development of various antibiotic formulations [24,25,26]. In general, a carrier is needed to generate spray-dried particles, and lactose monohydrate (LM; Figure 1b) is widely used to this end. LM is highly biocompatible, stable, cost-effective, and has excellent flowability [27].

Several studies have reported the production of spray-dried cipro particles, particularly using the salt form cipro hydrochloride (cipro HCl). For instance, biodegradable microparticles of poly(D,L-lactide-co-glycolide) (PLGA) loaded with cipro HCl have been produced via spray drying, achieving high drug encapsulation efficiency (90.5−105.5%) without significant interactions between the drug and the polymer matrix [28]. Additionally, excipients such as polyvinyl alcohol (PVA), L-leucine (LEU), and hydroxypropyl-β-cyclodextrin (CD) have been shown to modulate the physicochemical properties and stability of spray-dried cipro particles [29,30]. Notably, PVA/cipro HCl spray-dried particles demonstrated significantly reduced particle size, whereas LEU/cipro HCl particles exhibited rapid drug release [29]. Stability testing further revealed that LEU/cipro HCl spray-dried particles had the lowest aggregation and the highest stability among the tested formulations (spray-dried cipro HCl, PVA/cipro HCl, LEU/cipro HCl, CD/cipro HCl, and PVA/LEU/CD/cipro HCl) [30].

In general, the literature primarily employs organic solvents such as ethanol and/or acetone during spray drying of cipro, which contradicts the principles of green chemistry [31]. One notable exception is a study that used only water, cipro HCl, and lactose monohydrate to produce cipro HCl spray-dried microparticles via a simple green spray-drying process. The resultant microparticles exhibited good flowability and a high production yield (81.5%) [32]. Nevertheless, this study used cipro HCl rather than cipro. Although spray-dried cipro particles have been successfully prepared using aqueous PVA solutions, the drug concentration was limited to 10 wt.% [33].

Additionally, none of the above studies included antibacterial testing. A few studies have evaluated antibacterial activity, however, reporting that cipro HCl spray-dried particles containing 50% (*w*/*w*) mannitol had potent antibacterial activity against *S. aureus*, *P. aeruginosa*, and *Streptococcus pyogenes* [34]. Similarly, cipro-loaded dextran and chitosan SD microparticles with various surface modifiers exhibited antibacterial activity against *P. aeruginosa* and *S. aureus* while showing low toxicity to lung epithelial cells [35]. Cipro-loaded glutaraldehyde cross-linked SD gelatin microparticles exhibited MIC values comparable to free cipro against *S. aureus* and *Escherichia coli*, although the maximum explored drug concentration was only 5 wt.% [36]. The antibacterial tests in these studies primarily employed conventional methods, such as disc diffusion [34] and microdilution assays [35,36].

This study aims to fill some of the gaps left in previous research, including the reliance on complex excipients or organic solvents, low cipro concentrations, and the absence of antibacterial testing or reliance on conventional testing methods. We set out to achieve this by developing microparticles composed solely of lactose and cipro, without the use of organic solvents or additional excipients, through a single green spray-drying process. The focus was on achieving the highest possible cipro concentration and evaluating antibacterial activity through advanced isothermal calorimetry, which allows dynamic time-resolved assessment and comparison of bacterial metabolic responses to free cipro and cipro-loaded microparticles.

## 2. Materials and Methods

### 2.1. Materials

Materials were obtained as follows: ciprofloxacin (Fisher Scientific, Loughborough, UK); *P. aeruginosa* (NCTC 10662, Sigma-Aldrich, Buchs, Switzerland); *S. aureus* (WDCM 00032 Vitroids, Sigma-Aldrich, Buchs, Switzerland); lactose monohydrate (C_12_H_22_O_11_·H_2_O, MW 360.30 g/mol, Fisher Scientific, Loughborough, UK); phosphate-buffered saline (PBS) tablets (Sigma-Aldrich, Dorset, UK); acetic acid (AA, ≥99.7%, Fisher Scientific, Loughborough, UK); HCl (36.5–38%, Sigma-Aldrich, Dorset, UK); ethanol (≥99.8%, Sigma-Aldrich, Dorset, UK); glycerol (Fisher Scientific, Loughborough, UK); tryptic soy agar (TSA, Fisher Scientific, Loughborough, UK); tryptic soy broth (TSB, Fisher Scientific, Loughborough, UK); and deionized water (Purite, Triple Red Limited, Wycombe, UK).

### 2.2. Preparation of Spray Drying Solutions

After initial optimization, the base formulation for spray drying was set as 0.5% *w*/*v* LM in a mixture of water and AA at a ratio of 199:1 *v*/*v*. Cipro was added into the solutions at concentrations of 1, 10, 20, 40, and 50% cipro (*w*/*w*, relative to the total weight of cipro and LM).

### 2.3. Production of SD Particles

Solutions were spray dried on a B-290 Mini Spray-Dryer (Büchi Labortechnik AG, Flawil, Switzerland), using an inlet temperature of 200 °C, an aspirator rate of 85%, and a pump rate of 15%. The outlet temperature fluctuated between 40 and 60 °C.

### 2.4. Characterization

#### 2.4.1. Scanning Electron Microscopy (SEM)

A bench-top SEM (Phenom Pro, Thermo Fisher Scientific, Eindhoven, Netherlands) was employed to study the morphology of the SD particles. SEM images were collected after samples were sputter coated with graphite. For each formulation, 200 particles from different images were selected and analyzed with the ImageJ 1.52a software (National Institutes of Health, Bethesda, MD, USA). The size results are presented as mean ± standard deviation (SD). Size distributions were plotted using Origin 2022b (OriginLab Corporation, Northampton, MA, USA). Paired comparison (Tukey) was employed for statistical analysis, and statistical significance is indicated by asterisks, with *p*  ≤ 0.05 considered significant.

#### 2.4.2. X-Ray Diffraction (XRD)

A Rigaku Miniflex 600 diffractometer (Rigaku Corporation, Tokyo, Japan) supplied with Cu Kα radiation (λ = 1.5418 Å) was used for XRD analysis, which was performed at 40 kV and 15 mA over the 2θ range from 3 to 40° at 5°/min.

#### 2.4.3. Fourier-Transform Infrared Spectroscopy (FTIR)

A PerkinElmer Spectrum 100 FTIR spectrometer (PerkinElmer Inc., Waltham, MA, USA) was used to collect spectra (four scans) in attenuated total reflectance mode, within the range of 650–4000 cm^−1^ and at a resolution of 1 cm^−1^.

#### 2.4.4. Thermogravimetric Analysis (TGA)

Samples were heated from 40 to 500 °C at a rate of 10 °C/min on a TA Instruments Discovery TGA instrument (TA Instruments, New Castle, DE, USA), under a nitrogen gas flow rate of 25 mL/min. Analysis was performed using the TA Trios software (version 5.3.1, TA Instruments).

#### 2.4.5. Differential Scanning Calorimetry (DSC)

Samples were analyzed using a TA Instruments Q2000 DSC (TA Instruments, New Castle, DE, USA) under a nitrogen gas flow rate of 50 mL/min. Samples were heated at 10 °C/min from 0 °C to the final temperature, which was determined based on the TGA data and set to ensure no degradation occurred inside the DSC.

#### 2.4.6. Drug-Loading Determination

A total of 5 mg of each sample was dissolved in 10 mL of 0.1 M HCl to determine the content of cipro [37]. These solutions were stirred at 37 °C overnight, followed by centrifugation at 10,000 rpm for 10 min. Finally, the cipro concentration was determined from the UV absorbance of the supernatant, quantified with a Spectramax M2e instrument (Molecular Devices, San Jose, CA, USA) at a wavelength of 277 nm, using a linear calibration curve of cipro in 0.1 M HCl (0.5–15 μg/mL, R^2^ = 0.9999). The drug content and entrapment efficiency were calculated according to Equations (1) and (2):

Cipro content (%) = (mass of cipro in particles/mass of particles) × 100(1)

Entrapment efficiency (%) = (cipro content/theoretical content) × 100(2)

#### 2.4.7. Drug Release Assays

Samples were accurately weighed and transferred to a length of sealed dialysis tubing (BioDesign, 3500 Da MWCO, Fisher Scientific), which was then immersed in 200 mL of PBS (pH 7.4) and shaken in an incubator (50 rpm, 37 °C). For each formulation, sufficient mass was added to ensure a maximum cipro concentration of <5 μg/mL (calculated from the drug loading), thereby ensuring sink conditions were maintained. Periodically, 2 mL of the release medium was withdrawn and replaced with 2 mL of fresh preheated PBS. Cipro’s documented aqueous solubility is 30 mg/mL at 20 °C at pH 11 [33], and hence the maximum concentration possible in release experiments (5 μg/mL) was 6000 times lower than the solubility limit. The removed aliquots were analyzed on a Spectramax M2e instrument (Molecular Devices) at a wavelength of 270 nm, and the cipro concentration was calculated using a calibration curve in PBS (0.5–15 μg/mL, R^2^ = 0.9994), with results expressed as % cumulative release relative to the initial mass of cipro loaded in the particles. Analysis was performed in triplicate and results reported as mean ± SD. The results were fitted with the Korsmeyer–Peppas model (Equation (3)) to evaluate the release kinetics [33,38]:

M_t_/M_∞_ = kt^n^ (3)

where M_t_/M_∞_ is the fraction of drug released at time t; k is the rate constant; and n is an exponent that characterizes the release mechanism.

#### 2.4.8. Antibacterial Assays

##### Bacterial Culture

Both *P. aeruginosa* and *S. aureus* were cultured using identical procedures. Initially, a bacteria pellet was dispersed and vortexed in 1 mL of TSB (first generation), which was then transferred to and grown in 40 mL TSB in an incubator (37 °C) for 24 h. Following incubation, the resultant culture was vortexed, and 1 mL of the culture was transferred to 40 mL of fresh TSB for subculture (second generation). After subculture for two generations (always vortexing before subculture), 5 mL of the overnight culture from the last generation was added to 250 mL of fresh TSB broth and incubated at 37 °C. A 1 mL sample of the culture suspension was withdrawn, vortexed, and its optical density (OD) measured at a wavelength of 600 nm using a spectrophotometer (CO8000 cell-density meter, WPA Biowave, Cambridge, UK). Once the OD reading fell between 0.4 and 0.7, indicating the bacteria were in the exponential growth phase, the culture was processed for freezing.

In this processing, 0.1 mL of culture suspension was transferred into sterile tubes with 0.9 mL of PBS and labeled as 10^−1^. This solution was then serially diluted tenfold to obtain dilutions from 10^−2^ to 10^−7^. Subsequently, 50 μL from each dilution was inoculated onto TSA plates and spread with a fresh L-shaped hockey spreader. After 24 h of incubation, plates containing a countable number of colonies (ranging from 2 to 300 colonies) were selected, and the colonies were counted to establish the colony-forming units per milliliter (CFU/mL). This was performed for each dilution in triplicate. The final dilution chosen to count was 10^−5^ (10^−1^–10^−4^ had too many colonies). After counting the colonies in the 10^−5^ plates, the final inoculum calculated was 10^8^ CFU/mL for both *P. aeruginosa* and *S. aureus.*

The remaining liquid culture, with OD between 0.4 and 0.7, was centrifuged at 9500 rpm at 4 °C for 10 min, resulting in a pellet. The supernatant was discarded, and the tubes were refilled with PBS, in which the pellet was re-suspended and centrifuged. The PBS re-suspension and centrifugation processes were repeated 3 times. Finally, the pellet was resuspended in 20% glycerol, transferred into 2 mL cryovials and frozen at −80 °C. For each step, vortexing was necessary, and TSA plates were prepared for streaking to ensure purity control (without signs of contamination).

##### Antibacterial Efficacy of Cipro

The determination of the minimum heat inhibitory concentration (MHIC) of free cipro against *P. aeruginosa* and *S. aureus* was conducted in vitro using isothermal calorimetry. The MHIC was defined as the lowest concentration of antibiotic that did not lead to heat-flow production in the calorimeter within 24 h. To prepare the stock solution, free cipro was dissolved in 200 mL of 0.5% *v*/*v* aqueous AA to achieve a concentration of 1000 μg/mL. Solutions with concentrations of 100 μg/mL, 10 μg/mL, and 1 μg/mL of cipro were then obtained by diluting the stock solution with sterile water and filtering through a 0.22 μm filter. Different solutions were prepared for *P. aeruginosa* (NCTC 10662) and *S. aureus* (WDCM 00032 Vitroids) (Supporting Information, Tables S1 and S2). In total, 2 mL TSB (37 °C), cipro solution, and sterile water were pipetted into a calorimetric ampoule (glass, 3 mL). Bacterial culture (0.03 mL) was added to achieve a total volume of 3 mL. The ampoule was then sealed, vortexed, and transferred to a thermal activity monitor (TAM; TA Instruments Ltd., New Castle, DE, USA) where it was allowed to equilibrate for 30 min at 37 °C. Data were then recorded with the Digitam 4.1 software (amplifier setting 3000 μW). The isothermal calorimetry results are presented as curves of heat flow (μW) vs. time (h). Control groups comprised *P. aeruginosa*/*S. aureus* without cipro. Each concentration was tested in triplicate, and the resultant curves were either overlapped or were very close to each other. Therefore, one representative dataset is reported. The inoculation size of the bacteria was 10^6^ CFU/mL.

##### Antibacterial Activity of Cipro-Loaded SD Particles

The in vitro MHICs of SD particles against *P. aeruginosa* and *S. aureus* were also evaluated by TAM. To prepare particle suspensions with different concentrations, particle stock solutions (1000, 100, 10 μg/mL) were initially prepared and filtered through a 0.22 μm filter. TSB (2 mL, 37 °C), bacterial inoculum (0.03 mL), particle stock suspension, and water were then pipetted into ampoules to give a total volume of 3 mL. Subsequently, the ampoules were loaded into the TAM as described above. Control groups consisted of pure *P. aeruginosa* or *S. aureus*, and the blank formulation (spray dried from 0.5% *w*/*v* LM in 199:1 *v*/*v* water/AA; 300 μg/mL). Four concentrations were tested for each formulation, and each concentration was analyzed in triplicate. The resultant curves were either overlapped or very close to each other, and hence one representative dataset is reported. The results are presented as plots of heat flow (μW) vs. time (h). The inoculation size of the bacteria was 10^6^ CFU/mL.

## 3. Results and Discussion

### 3.1. Formulation Optimization

Based on previous findings [39], initial SD parameters were set to an inlet temperature of 200 °C, aspirator at 85%, and pump at 15%. Initially, water was the solvent, and the LM concentration was set at 0.5% *w*/*v*. This gave spherical particles with a mean size of 2.77 ± 0.75 μm (see Figure S1). Subsequently, various concentrations of cipro were added to the solution. However, when using water, the low solubility of cipro meant that the maximum drug loading was 1% *w*/*w* with respect to the total mass of solute. This produced spherical particles with a mean size of 3.03 ± 0.58 μm (see Figure S1). To achieve higher drug loading, the solvent was thus modified to a mixture of water and AA at 199:1 *v*/*v* since the solubility of cipro is higher at low pH [16].

**Figure 2:**
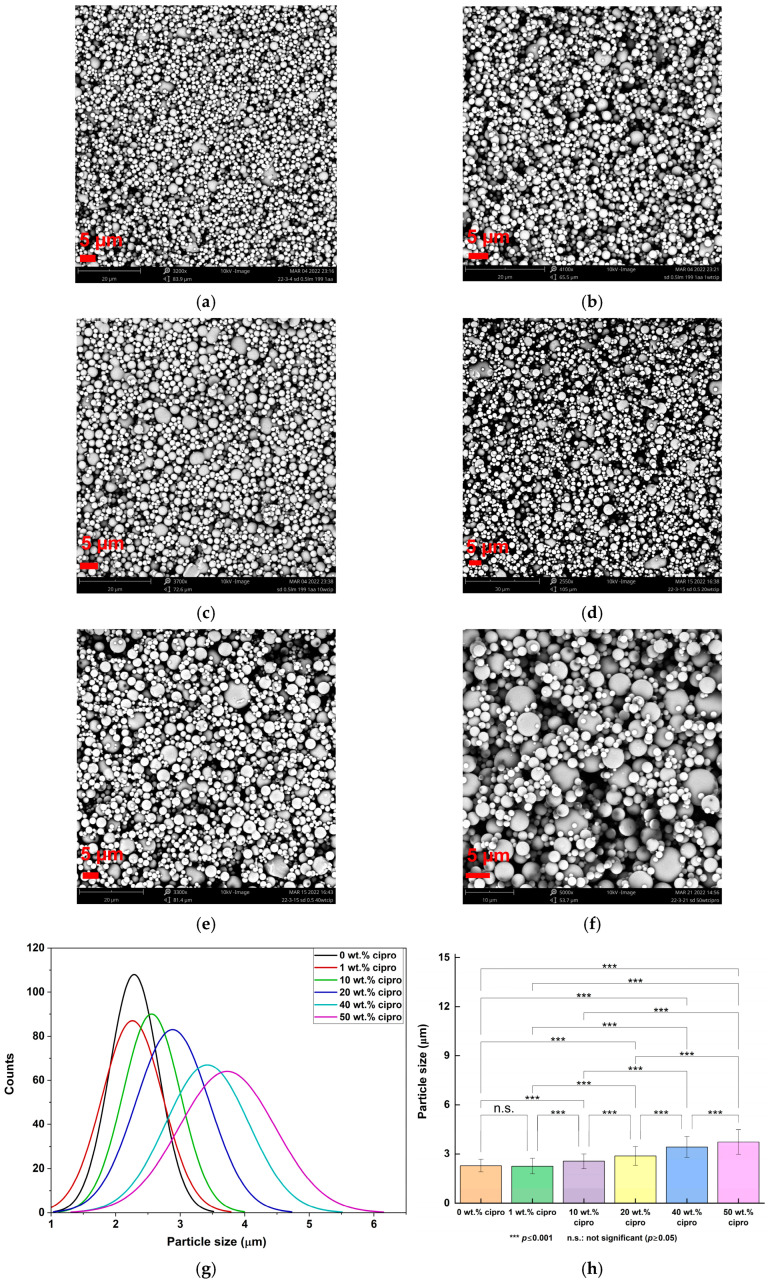
SEM images of particles prepared from (**a**) 0.5% *w*/*v* LM in water/AA, 199:1 *v*/*v*; (**b**) 1, (**c**) 10, (**d**) 20, (**e**) 40, (**f**) 50 wt. % cipro/0.5% *w*/*v* LM in water: AA, 199:1 *v*/*v*; (**g**) their size distribution curves and (**h**) statistical analysis of their particle sizes.

Using 0.5% *w*/*v* LM in water/AA = 199:1 *v*/*v* yielded uniform spherical particles (Figure 2) and enabled the drug loading to be increased to 50% *w*/*w* relative to the total weight of solute. All the particles exhibited a narrow size distribution, with extensive particle aggregation observed (Figure 2), as is typical for SD microparticles [32,33]. An increase in cipro content led to larger particle sizes, ranging from 2.26 ± 0.47 μm (1 wt. % cipro) to 3.73 ± 0.74 μm (50 wt. % cipro) (Table 1).

### 3.2. Characterization

#### 3.2.1. XRD

**Figure 3:**
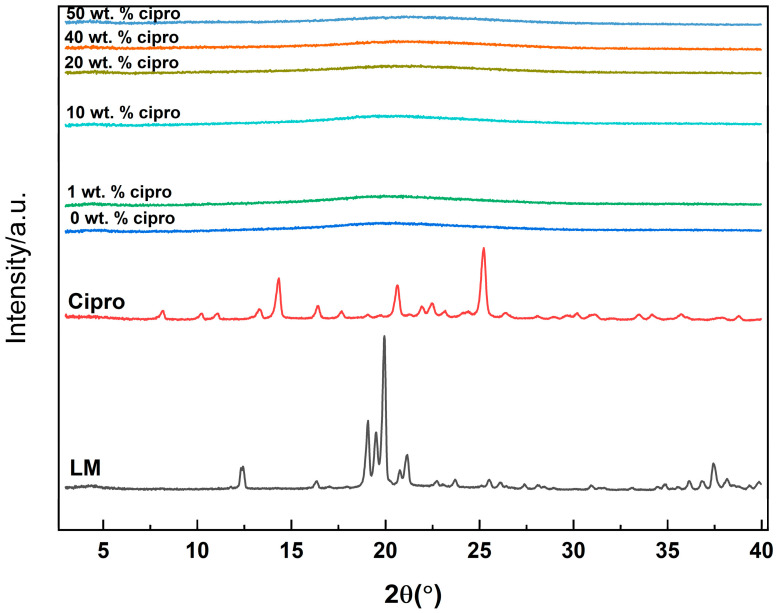
XRD patterns of the SD particles.

XRD patterns for the SD particles are presented in Figure 3. In contrast to the crystalline nature of raw cipro and LM, which are characterized by sharp Bragg reflections in their diffraction patterns, all SD powders exhibit only diffuse haloes. This indicates the absence of crystalline LM or cipro forming during the drying process. These findings suggest that both cipro and LM underwent conversion into the amorphous physical form through spray drying. The conversion to the amorphous form is advantageous as it enhances the solubility and dissolution rate of the drug, particularly beneficial for poorly water-soluble drugs like cipro [40]. The rapid solvent evaporation during spray drying typically prevents molecular ordering and crystallization, leading to a more disordered structure that may improve the bioavailability of the drug [41,42].

#### 3.2.2. FTIR

**Figure 4:**
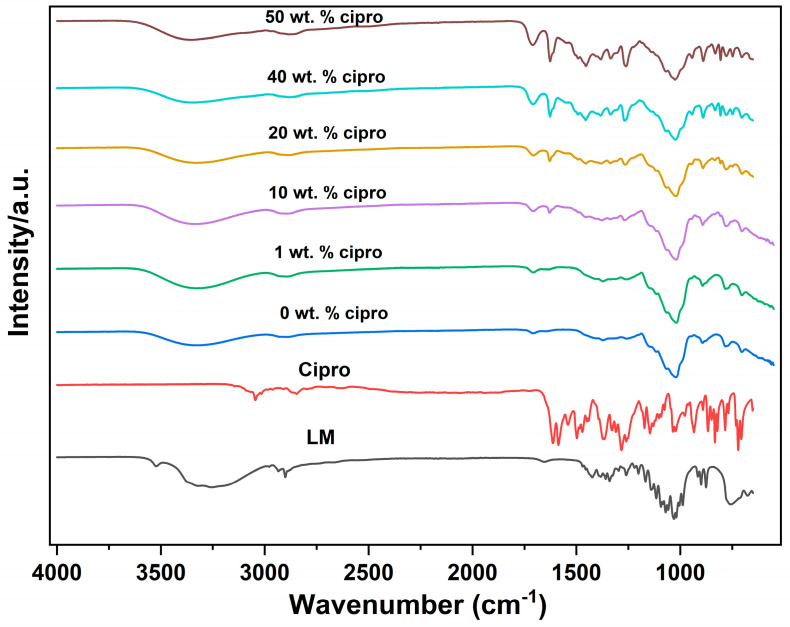
FT−IR spectra of the SD particles.

The IR spectra of the SD particles are presented in Figure 4. In the spectrum of cipro, bands at 3043 and 2851 cm^−1^ correspond to the C−H stretching vibration from the phenyl framework [43]. Cipro also shows distinct peaks at 1585 and 1542 cm^−1^, resulting from benzene-ring stretching vibrations [44], and a C=O stretch at 1612 cm^−1^. In the spectrum of LM, a sharp, distinct, O−H stretch (of water molecules) is observed at 3529 cm^−1^. The shape and location of this peak are indicative of constrained water in the crystal lattice [45]. The band at 1655 cm^−1^ is the delta bend of water molecules [45]. In addition, characteristic doublet peaks at 1036 and 1022 cm^−1^ correspond to C−C stretching [46] and a band at 1200−1060 cm⁻^1^ is attributed to the asymmetric bending of C−O−C in glucose and galactose [46].

For the SD formulations, the broad band between 3600 and 3000 cm^−1^ corresponds to the lactose O−H groups. The sharp OH stretch at 3529 cm^−1^ is missing, however, indicating the absence of crystal lattice water in the SD particles [47]. Successful drug loading is confirmed by the presence of characteristic cipro absorption bands. For example, the peak at approximately 1612 cm^−1^, attributed to the C=O of cipro, is shifted to higher wavenumbers (1625 cm^−1^) after drug loading but remains observable in the spectra of the SD formulations [48]. The peak shift may result from potential hydrogen bonding or other intermolecular interactions between the drug and LM [49]. With an increase in drug content, the distinctive cipro peaks become sharper and more pronounced, indicative of higher drug loading.

#### 3.2.3. Thermal Analysis

TGA curves (Figure S2) of the SD particles show similar weight loss trends to LM: firstly, water loss, followed by the decomposition of the formulations. The onset temperature of decomposition varies. Decomposition of the formulations starts at lower temperatures than for the raw materials, and SD formulations with higher cipro contents are more likely to decompose at lower temperatures, but the trend is not completely clear.

DSC curves are displayed in Figure S3. The SD formulations manifest shallow broad endotherms in the range of 30−70 °C, probably due to water loss. The SD particles degrade at temperatures below the cipro melting point, so no evidence for physical form may be directly inferred from the DSC experiments.

#### 3.2.4. Drug Loading

As detailed in Table 2, the SD particles achieved a maximum drug loading of 50%. The SD particles have encapsulation efficiency (EE) values close to 100%, indicating an efficient drying process. In some cases, the values lie a little below 100% (10, 20 wt. % cipro), which can be attributed to the sample collection process, during which a considerable amount of sample powder adhered to the collector wall. Conversely, EE values exceeding 100% might be indicative of surface adsorption of the drug leading to an overestimation of EE [50]. This could also be attributed to lactose loss during the spray-drying process, such as through adhesion to the chamber walls or incomplete particle collection [51,52].

#### 3.2.5. Drug Release Profiles

**Figure 5:**
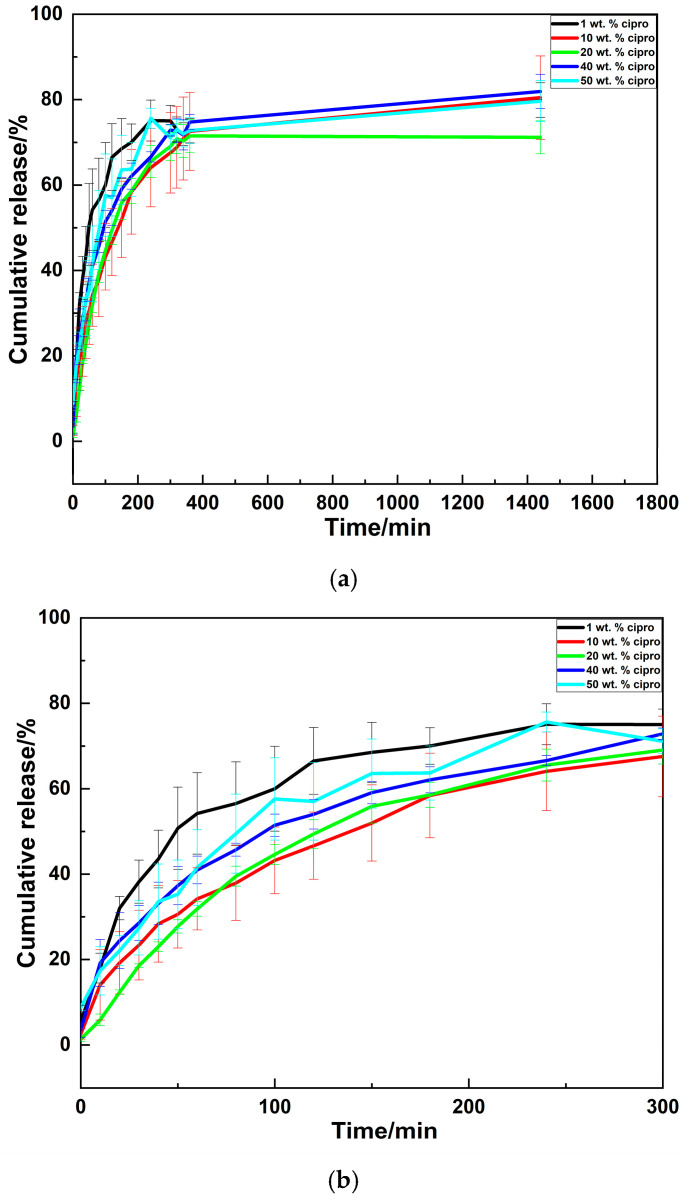
(**a**) Release profiles of the SD particles (mean ± SD, n = 3). (**b**) Enlarged view of the first 5 h.

Drug-release results (Figure 5) reveal similar release profiles for all the SD particles, characterized by initial rapid release within the first few hours, followed by a slower release phase. However, it is important to note that the initial fast release rates observed in vitro may be partly attributed to the sink conditions employed. This setup will not entirely reflect the in vivo drug-release scenario since the liquid volume used in the release experiment was 200 mL—likely much larger than the actual volume at infection sites, such as in ear infections (where the ear canal volume ranges from 0.6–1.8 mL in adults and is even smaller in children [53]). Therefore, the actual in vivo drug release may differ, occurring more slowly owing to the smaller fluid volumes. Further in vivo studies or volume-specific in vitro models are necessary to verify whether the release profiles are consistent with the intended applications. Additionally, as discussed in Section 3.2.4, the possible presence of surface-adsorbed drug molecules may also contribute to the initial burst release. It is observed that the SD particles do not achieve complete drug release, with maximum percentages of 70–81% noted after 24 h. This may stem from strong interactions between the cipro and LM [54], which limits the amount of drug which can exit the formulations.

The cumulative drug-release data were fitted using the Korsmeyer–Peppas model [55,56,57,58]. This model is applicable only when the M_t_/M_∞_ value is less than 0.60 [38,59]. Therefore, the data from the first hour of release were employed for modeling (Figure S4). The kinetic parameters are presented in Table 3.

In the context of spherical particles, three distinct in vitro release mechanisms are recognized based on the value of the exponent n: Fickian release (n ≤ 0.43, indicative of diffusion-controlled release), non-Fickian release (0.43 < n < 0.85, suggestive of anomalous transport), and case-II transport (n ≥ 0.85, associated with disintegration and relaxation-controlled release) [60]. It is observed that most of the SD formulations exhibit non-Fickian release, suggesting that drug release is governed by a combination of drug diffusion and relaxation. However, for 20 wt. % cipro (n = 0.945) and 40 wt. % cipro (n = 0.423), the release mechanisms appeared to align with case-II transport and diffusion-controlled release, respectively. In the case of the 20 wt.% cipro, the observed lower rate of release (k) is attributed to the case-II transport mechanism, where drug release is controlled by the disintegration or swelling of the particles rather than diffusion [60]. The observed differences in release mechanisms at 20 wt.% and 40 wt.% cipro could be influenced by variations in particle structure and drug distribution. Higher drug concentrations may lead to changes in particle morphology, porosity, or localized drug clustering, which can collectively affect diffusion pathways and release kinetics [61,62,63,64].

#### 3.2.6. Antibacterial Activities

##### Studies with Cipro

The MHIC values of cipro against *P. aeruginosa* and *S. aureus* were determined by isothermal calorimetry (Figures S5 and S6). Compared to traditional static antibacterial methods, such as disk diffusion or broth dilution, isothermal calorimetry has proven to be a highly effective tool for evaluating antibacterial activity [65,66,67,68]. This technique enables real-time, dynamic monitoring of bacterial metabolic responses to antibiotics, offering a more comprehensive understanding of antimicrobial effects [69]. Besides, it is highly sensitive and quantitative, capable of detecting subtle changes in bacterial activity even at low drug concentrations [69]. Additionally, isothermal calorimetry is simple, non-invasive, and label-free, requiring no complex reagents [68]. It can assess both inhibitory and bactericidal effects, providing valuable insights into the action mechanism of drugs [70,71]. Furthermore, because it is completely passive, the contents of the ampoule (e.g., media and bacteria) can be further analyzed using any desired method after the evaluation [65].

Both MHIC and MIC measure the lowest inhibition concentrations of antimicrobial agents, but their methodologies differ. MIC, determined through traditional methods like broth dilution or agar diffusion, provides a static endpoint by observing bacterial growth inhibition [72]. In contrast, MHIC uses isothermal calorimetry to monitor bacterial metabolic activity in real time, offering dynamic insights into bacterial responses to the drug [69]. MHIC also reveals more detailed information on bacterial metabolism and drug action mechanisms and is more sensitive, though it requires specialized equipment [65].

The complex shape of the growth curve reflects organism growth in an oxygen-limited medium (the sealed ampoule restricts oxygen to that which is dissolved in the medium and present in the headspace) [73]. Initially, the exponential phase represents aerobic metabolism (first peak), followed by a transition to anaerobic metabolism at around 5 h, initiating a second exponential growth phase (second peak). Successive peaks and troughs suggest the sequential utilization of major nutrients in the medium [73]. However, as nutrients are depleted or environmental conditions worsen due to metabolite accumulation, the heat-producing reactions cease, causing the power signal to return to the baseline [73].

Comparison with the control growth curves (GCs) reveals that the presence of cipro delays bacterial growth for both *P. aeruginosa* and *S. aureus*. Furthermore, when the drug concentration reaches or exceeds 0.5 μg/mL (*P. aeruginosa*) and 0.8 μg/mL (*S. aureus*), no growth is observed, resulting in a zero-power signal. These observations indicate an MHIC of 0.5 μg/mL for *P. aeruginosa* and 0.8 μg/mL for *S. aureus*.

##### Antibacterial Effect of Cipro-Loaded SD Particles

The MHIC values of cipro-loaded SD particles against *P. aeruginosa* and *S. aureus* were also determined by isothermal calorimetry. Both respond to the blank formulation, as indicated by the respective increases and decreases in heat-flow production compared to the GC. For *P. aeruginosa*, the increased heat production suggests a higher number of organisms in the presence of the blank formulation. This arises due to the presence of lactose in the particles, which is known to provide nutrients for *P. aeruginosa* growth [74]. In contrast, for *S. aureus*, decreased heat production indicates a lower number of organisms with the blank formulation, potentially due to the antimicrobial properties of residual AA in the formulation [75]. The presence of residual AA in the formulation can be inferred from both TGA and DSC analyses. In the TGA, after the loss of free water in the first stage (around 70–120 °C), 0 wt. % cipro exhibits a subtle, smooth mass loss, which may indicate the volatilization of residual AA. Correspondingly, a relatively flat endothermic peak is observed in the DSC curve within this temperature range, which may further suggest the presence of residual AA.

**Figure 6:**
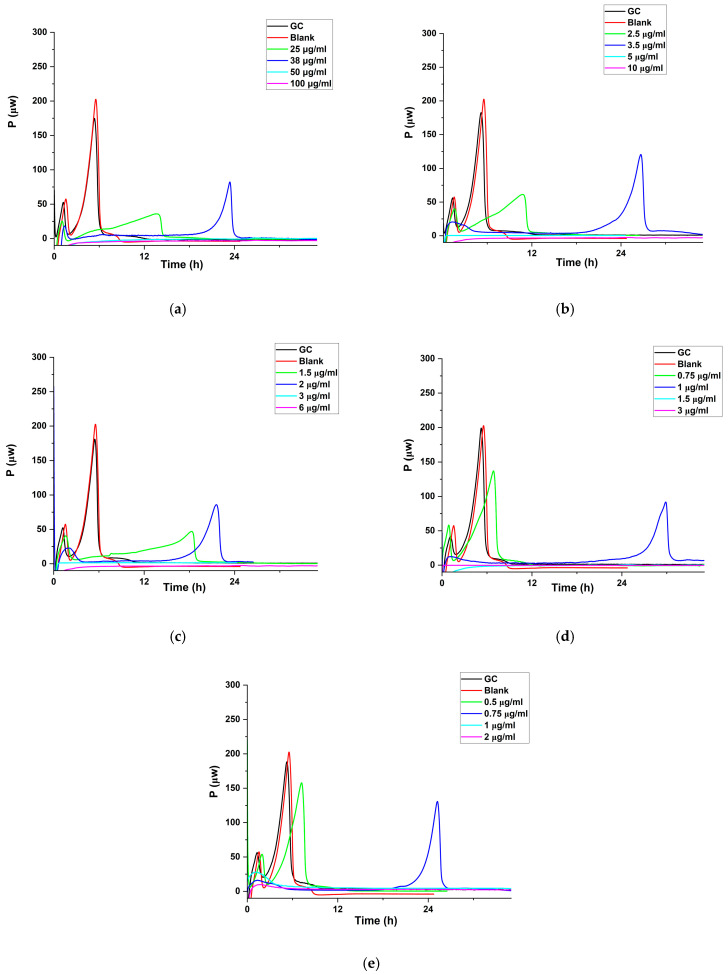
Heat production (μW) vs. time (h) for *P. aeruginosa* incubated with the blank formulation and different concentrations of cipro-loaded SD particles with (**a**) 1, (**b**) 10, (**c**) 20, (**d**) 40, and (**e**) 50 wt. % drug. Inoculation size was 10^6^ CFU/mL.

**Figure 7:**
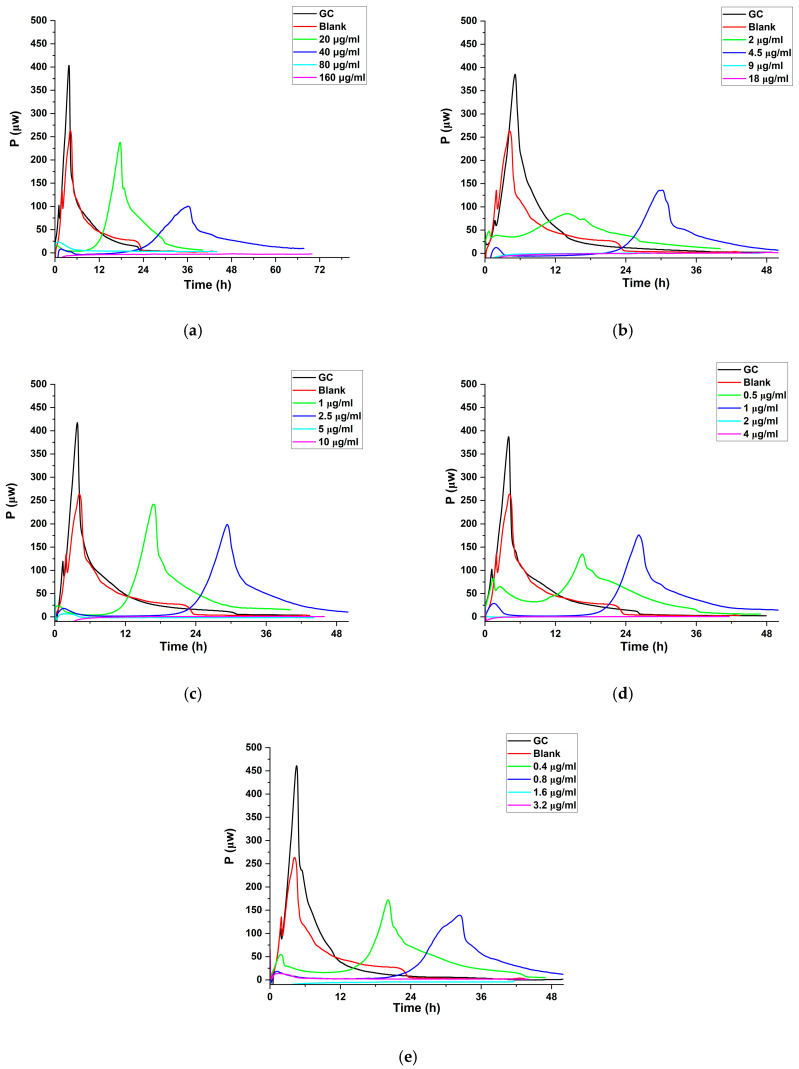
Heat production (μW) vs. time (h) for *S. aureus* incubated with the blank formulation and different concentrations of cipro-loaded SD particles containing (**a**) 1, (**b**) 10, (**c**) 20, (**d**) 40, and (**e**) 50 wt. % cipro. Inoculation size was 10^6^ CFU/mL.

In the SD formulations, cipro retains its antibacterial efficacy, as evidenced by the concentration-dependent delayed growth of both *P. aeruginosa* and *S. aureus* compared to the untreated control GC (Figure 6 and Figure 7). The MHIC values of cipro-loaded SD particles against *P. aeruginosa* and *S. aureus* are summarized in Table 4. SD formulations with higher drug loading exhibit lower MHIC values. The cipro-loaded SD formulations show an antibacterial effect comparable to free cipro, as is clear if the MHIC values of the formulations are normalized to their cipro content. It is evident that spray drying and the interactions between cipro and lactose do not affect the antibacterial efficacy of cipro, which aligns with previous findings [35,76].

In previous studies on cipro or cipro HCl SD particles, most formulations—except for cipro-loaded PVA microparticles—exhibited collapsed or rough particle surfaces [28,29,30,31,32,33,34,35,36]. In contrast, the particles produced in this study maintained a smooth, spherical morphology. Additionally, while cipro-loaded lactose particles produced through simple, green spray-drying methods typically form crystalline structures [32], the particles in this study were entirely amorphous. Similar crystalline tendencies have been reported for LEU/cipro HCl and PVA/LEU/CD/cipro HCl SD particles [30]. In terms of the drug-release behavior, previous studies have shown that cipro-loaded gelatin microparticles exhibited a release pattern similar to our formulations, characterized by an initial burst release within the first six hours, followed by a slower sustained-release phase under sink conditions [36]. Other formulations demonstrated varying release profiles, such as rapid release within 3 min (e.g., LEU/cipro HCl SD particles) [30] or sustained release over 24 h (e.g., cipro-PVA SD particles) [33].

The SD cipro particles developed in this study demonstrated antibacterial efficacy comparable to that of free cipro, which is consistent with findings reported in the literature [34,35,36]. For example, the efficacy of cipro in SD particles has been demonstrated for mannitol/cipro HCl [34] and cipro/chitosan/dextran SD microparticles with various surface modifiers [35], as well as cipro-loaded glutaraldehyde crosslinked gelatin particles [36]. However, the particles developed in this study offer several advantages over those in the literature. These include markedly higher cipro loading (up to 50 wt.% compared to the highest 38–40 wt.% reported in the literature [35]) and our use of a simple, green, aqueous-based spray-drying process. This approach eliminates the need for complex excipients or organic solvent, simplifying production and enhancing scalability. Furthermore, this work is the first to explore the efficacy of cipro-loaded SD particles using time-resolved isothermal calorimetry, which reveals that bacterial growth can be fully inhibited for at least 36 h at concentrations above the MHIC. These factors underscore the potential of the SD cipro particles as a practical and effective formulation for antibacterial therapies.

## 4. Conclusions

Following a series of optimization experiments, lactose-based microparticles were successfully prepared through spray drying (SD). The particles were formulated with cipro loadings ranging from 1 to 50% *w*/*w*. SEM analysis confirmed the particles to have spherical morphology and sizes between 2.26 and 3.73 μm. Both cipro and lactose were rendered amorphous through the spray-drying process. The SD particles exhibited rapid cipro release within 5 h, suggesting potential efficacy in treating acute bacterial infections. Furthermore, the particles demonstrated concentration-dependent antibacterial activity against both *P. aeruginosa* and *S. aureus*, significantly inhibiting bacterial growth. Growth could be completely prevented for at least 48 h, using concentrations above the maximum heat-inhibitory concentration of the particles.

## Figures and Tables

**Table 1:** Mean particle sizes prepared with 1, 10, 20, 40, 50 wt. % cipro (0.5% *w*/*v* LM, water/AA = 199:1 *v*/*v*).

Formulation	Mean Particle Size (μm)
0.5% *w*/*v* LM, water/AA = 199:1 *v*/*v*	2.29 ± 0.38
1 wt. % cipro	2.26 ± 0.47
10 wt. % cipro	2.56 ± 0.44
20 wt. % cipro	2.88 ± 0.57
40 wt. % cipro	3.42 ± 0.64
50 wt. % cipro	3.73 ± 0.74

**Table 2:** Drug loading and entrapment efficiency (EE) of SD particles.

Formulation	Drug Loading (%)	EE (%)
1 wt. % cipro	1.1 ± 0.0	107.0 ± 1.9
10 wt. % cipro	9.9 ± 0.3	98.6 ± 2.6
20 wt. % cipro	19.4 ± 0.2	96.9 ± 0.9
40 wt. % cipro	42.1 ± 0.5	105.2 ± 1.2
50 wt. % cipro	52.9 ± 1.6	105.7 ± 3.2

**Table 3:** Korsmeyer–Peppas fit parameters for the SD particles.

Formulations	n	k (min^−1^)	R^2^
1 wt. % cipro	0.603	0.048	0.981
10 wt. % cipro	0.499	0.044	0.996
20 wt. % cipro	0.945	0.007	0.994
40 wt. % cipro	0.423	0.070	0.990
50 wt. % cipro	0.485	0.054	0.982

**Table 4:** MHIC values of cipro-loaded SD particles against *P. aeruginosa* and *S. aureus*. MHIC values are of the particles, while the normalized values are adjusted for the cipro content.

Formulation	*P. aeruginosa*		*S. aureus*	
	MHIC of SD (μg particles/mL)	Normalized MHIC (μg Cipro/mL)	MHIC of SD (μg particles/mL)	Normalized MHIC (μg Cipro/mL)
1 wt. % cipro	50	0.54	80	0.86
10 wt. % cipro	5	0.49	9	0.89
20 wt. % cipro	3	0.58	5	0.97
40 wt. % cipro	1.5	0.63	2	0.84
50 wt. % cipro	1	0.53	1.6	0.85

## Data Availability

The data supporting the reported results are available from the authors upon request.

## Supplementary Materials

The following supporting information can be downloaded at https://www.mdpi.com/article/10.3390/pharmaceutics17030392/s1. Table S1: Solutions of cipro prepared for P. aeruginosa calorimetry. Table S2: Solutions of cipro prepared for S. aureus calorimetry. Figure S1: SEM images of particles prepared from (a) 0.5% *w*/*v* LM, (b) 0.5% *w*/*v* LM/1 wt. % cipro in water and (c) Size distribution curves of particles prepared from 0.5 % *w*/*v* LM and 1 wt. % cipro/0.5% *w*/*v* LM in water. Figure S2: (a) TGA curves and (b) enlarged view of the SD formulations. Figure S3: DSC curves of the SD formulations. Exo up. Figure S4: Fits of the Korsmeyer–Peppas model to the release data for the SD particles. Figure S5: Heat flow (μW) vs. time (h) curves of P. aeruginosa incubated with different concentrations of free cipro (GC: negative control growth curve). Inoculation size was 106 CFU/mL. Figure S6: Heat flow (μW) vs. time curves of (a) S. aureus incubated with different concentrations of free cipro and (b) enlarged view with 0.6, 0.7, 0.8 μg/mL of cipro (GC: negative control growth curve). Inoculation size was 106 CFU/mL.
