# Supplementary material for: Ciprofloxacin-Loaded Spray-Dried Lactose Particles: Formulation Optimization and Antibacterial Efficacy
Source: Pharmaceutics. 2025 Mar 20;17(3):392. doi: 10.3390/pharmaceutics17030392 (PMC11945521; doi:10.3390/pharmaceutics17030392)
Supplement: Supplementary file 1 [file pharmaceutics-17-00392-s001.zip › pharmaceutics-3490509-supplementary.pdf]

Table S1 Solutions of cipro prepared for *P. aeruginosa* calorimetry

Reagent	Volumes of reagent/organism (ml)					
Cipro (10 μg/mL)	0	0.06	0.12	0.135	0.15	0.3
Test organism	0.03	0.03	0.03	0.03	0.03	0.03
Sterile water	0.97	0.91	0.85	0.835	0.82	0.67
TSB	2	2	2	2	2	2
Total volume	3	3	3	3	3	3
Final drug concentration (μg/mL)	**0**	**0.2**	**0.4**	**0.45**	**0.5**	**1**

Table S2 Solutions of cipro prepared for *S. aureus* calorimetry

Reagent	Volumes of reagent/organism (ml)								
Cipro (10 μg/mL)	0	0.03	0.12	0.15	0.18	0.21	0.24	0.3	0.45
Test organism	0.03	0.03	0.03	0.03	0.03	0.03	0.03	0.03	0.03
Sterile water	0.97	0.94	0.85	0.82	0.79	0.76	0.73	0.67	0.52
TSB	2	2	2	2	2	2	2	2	2
Total volume	3	3	3	3	3	3	3	3	3
Final drug concentration (μg/mL)	**0**	**0.1**	**0.4**	**0.5**	**0.6**	**0.7**	**0.8**	**1**	**1.5**

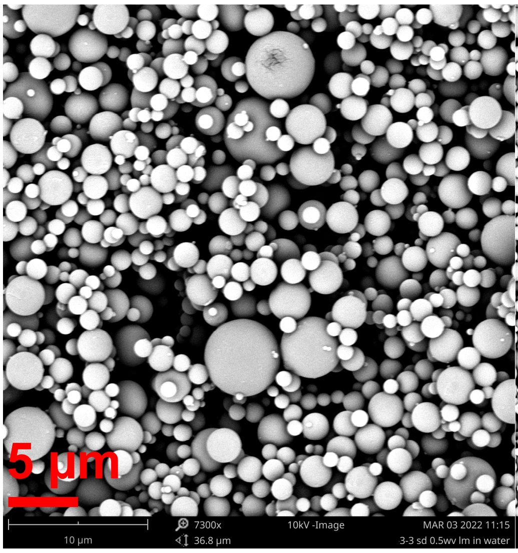

(**a**)

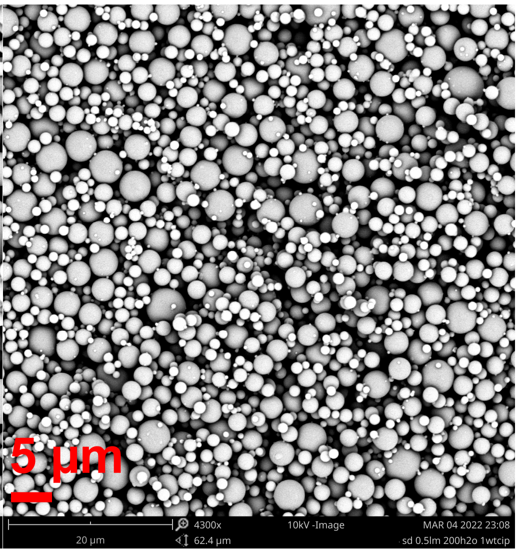

(**b**)

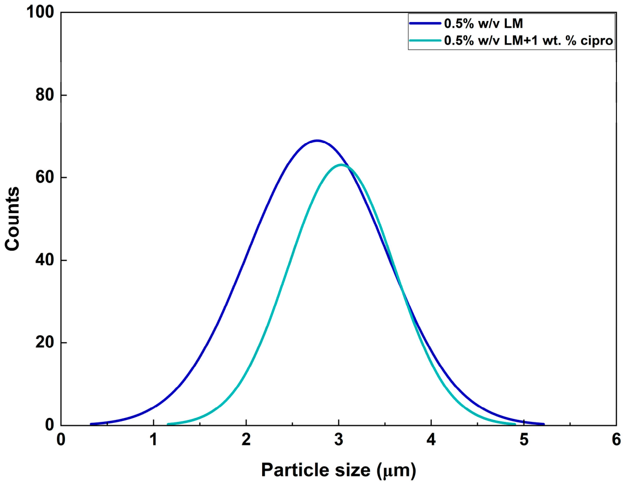

(**c**)

Figure S1 SEM images of particles prepared from **(a)** 0.5 % w/v LM, **(b)** 0.5% w/v LM / 1 wt. % cipro in water and **(c)** size distribution curves of particles prepared from 0.5 % w/v LM and 1 wt. % cipro / 0.5 % w/v LM in water.

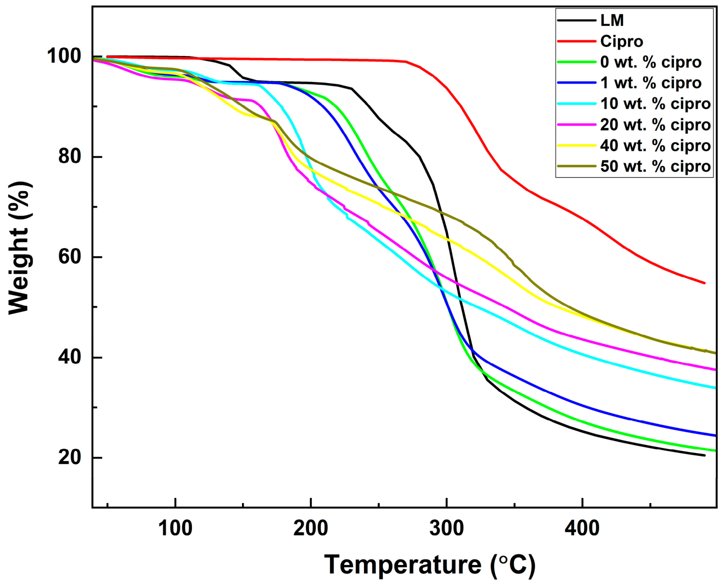

(**a**)

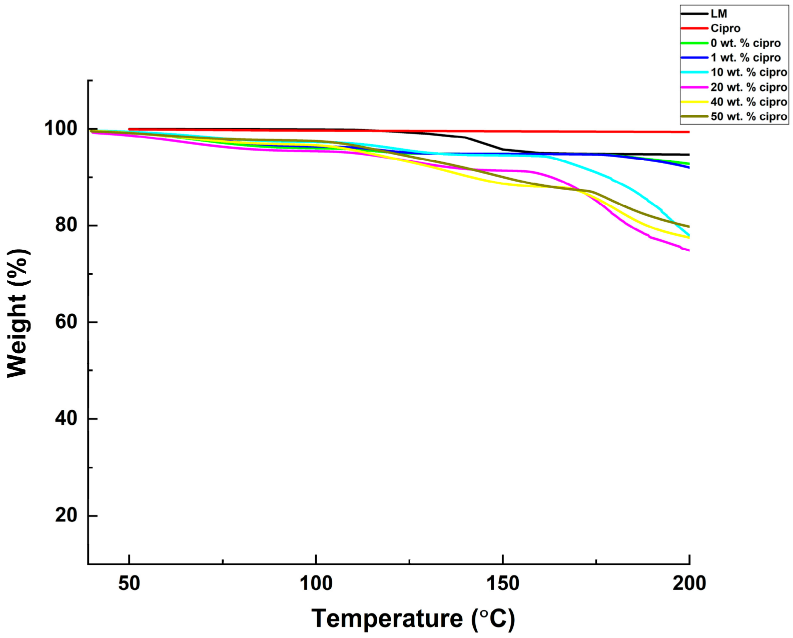

(**b**)

Figure S2 (a) TGA curves and **(b)** enlarged view of the SD formulations. Cipro decomposition begins above 268 °C. LM undergoes weight loss in three primary steps: loss of surface water (40-100 °C), loss of crystalline water (130~160 °C, with a loss of approximately 5% of the initial mass in agreement with the water content of lactose monohydrate), charring and decomposition of the lactose (above 220 °C). The SD formulations show similar weight loss trends to LM.

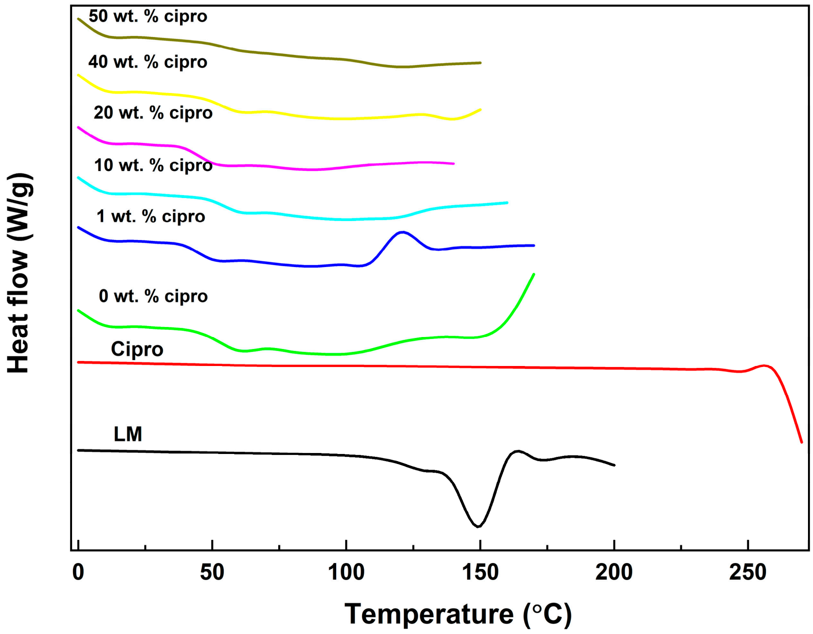

Figure S3 DSC curves of the SD formulations. Exo up. Due to the tendency of SD formulations to decompose at lower temperatures, the DSC traces of the SD formulations terminate at a lower temperature than the raw materials. Cipro exhibits a sharp endothermic melting peak at ca. 260 °C, confirming its crystalline nature. LM displays an endothermic event at 150 °C attributed to the loss of crystalline water. A small endothermic peak at ~170 °C might be associated with the melting of the remaining anhydrous lactose. The exothermic peak (160 - 180 °C) in the 0 wt.% SD formulation might be attributed to the transformation of amorphous lactose to a crystalline state during heating, mutarotation of α-lactose to β-lactose, or crystallization of unstable anhydrous and amorphous α-lactose into the crystalline β/α- compound. The exotherm (ca. 108 °C) in the 1 wt. % SD formulation might also be associated with the crystallization of anhydrous lactose. In terms of the physical form of the components of the SD particles, no significant insight may be gained from the data here, because the DSC traces were stopped before reaching the melting temperature of the LM or cipro.

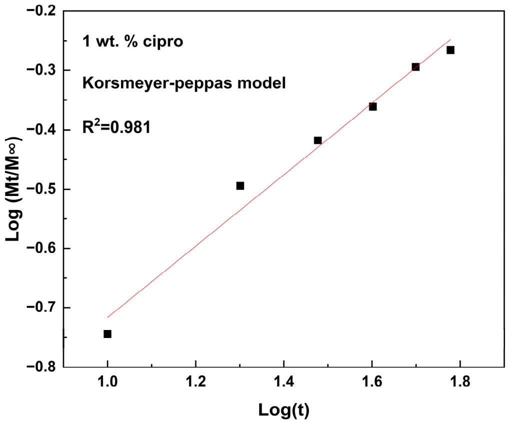

(**a**)

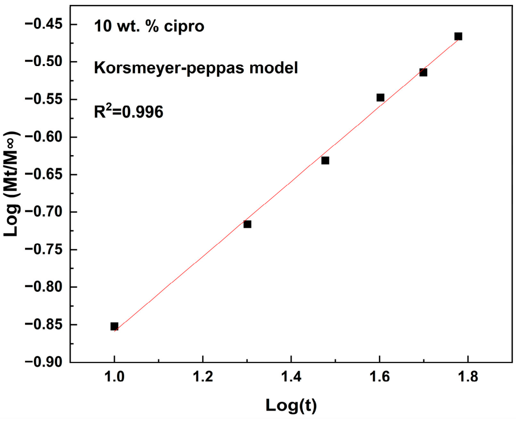

(**b**)

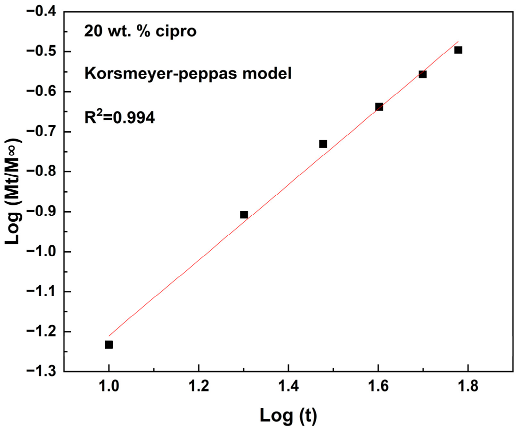

(**c**)

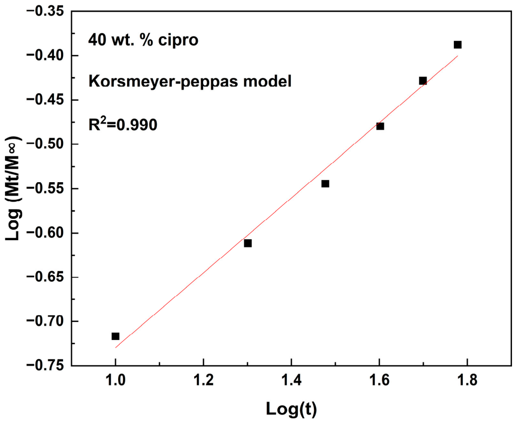

(**d**)

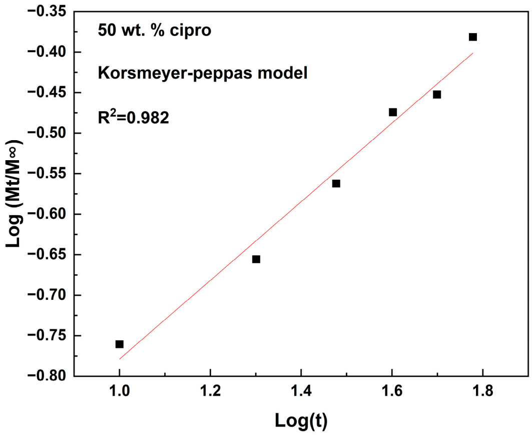

(**e**)

Figure S4 Fits of the Korsmeyer–Peppas model to the release data for the SD particles.

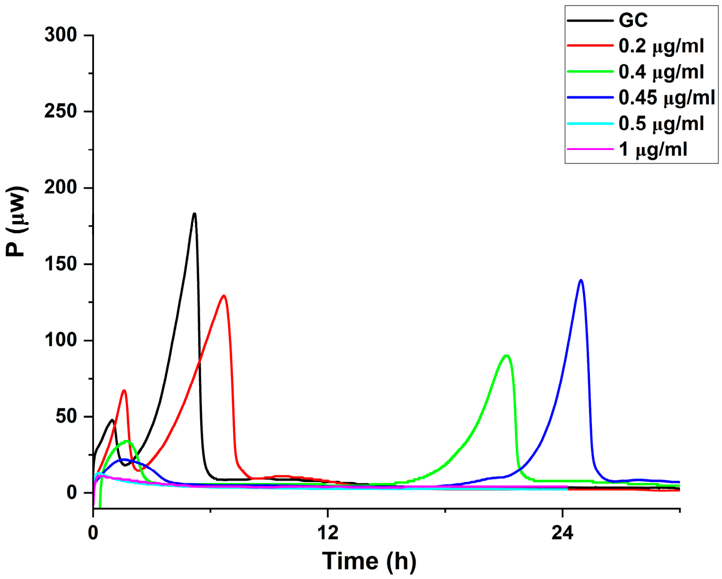

Figure S5 Heat flow (µW) vs time (h) curves of *P. aeruginosa* incubated with different concentrations of free cipro (GC: negative control growth curve). Inoculation size was 10^6 CFU/ml.

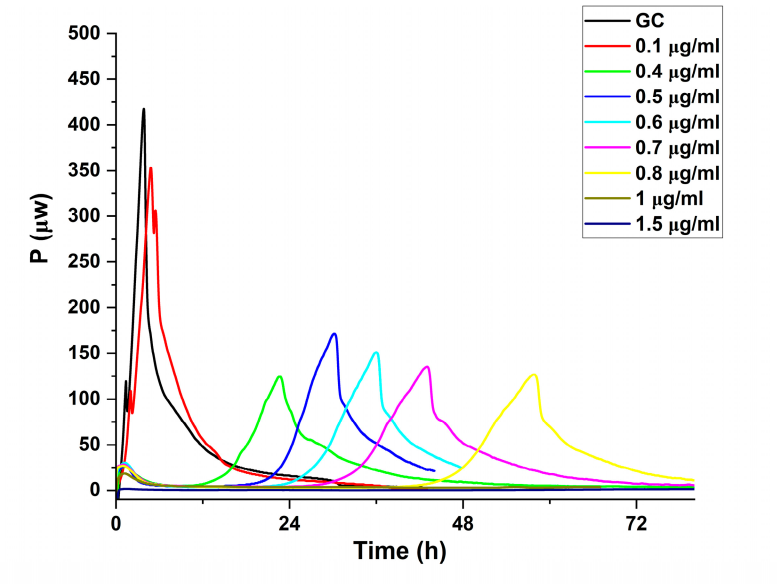

(**a**)

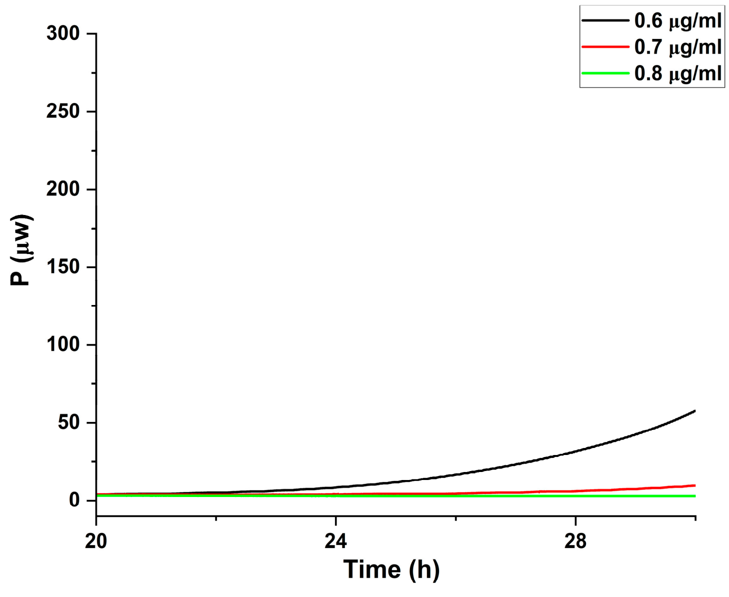

(**b**)

Figure S6 Heat flow (µW) vs time curves of **(a)** *S. aureus* incubated with different concentrations of free cipro and **(b)** enlarged view with 0.6, 0.7, 0.8 µg/ml of cipro (GC: negative control growth curve). Inoculation size was 10^6 CFU/ml.
